# Farm income and production impacts from the use of genetically modified (GM) crop technology 1996-2020

**DOI:** 10.1080/21645698.2022.2105626

**Published:** 2022-08-19

**Authors:** Graham Brookes

**Affiliations:** PG Economics, Hereford, United Kingdom

**Keywords:** Cost, genetically modified crops, income, production, yield

## Abstract

This paper updates previous estimates for the global value of using genetically modified (GM) crop technology in agriculture at the farm level. It examined impacts on yields, important variable costs of production, including the cost of the technology, direct farm (gross) income, and impacts on the production base of the main crops where the technology is used (soybeans, corn, cotton, and canola). Over the period 1996 to 2020, the economic benefits have been significant with farm incomes for those using the technology having increased by $261.3 billion US dollars. This equates to an average farm income gain across all GM crops grown in this period of about $112/hectare. In 2020, the farm income gains were $18.8 billion (average of $103/ha). The cumulative farm income gains have been divided 52% to farmers in developing countries and 48% to farmers in developed countries. Seventy-two percentage of the gains have derived from yield and production gains with the remaining 28% coming from cost savings. These yield and production gains have made important contributions to increasing global production levels of the four main crops, having, for example, added 330 million tonnes and 595 million tonnes respectively, to the global production of soybeans and maize since the introduction of the technology in the mid-1990s. In 2020, the extra global production of the four main crops in which GM technology is widely used (85 million tonnes), would have, if conventional production systems been used, required an additional 23.4 million ha of land to be planted to these crops. In terms of investment, for each extra dollar invested in GM crop seeds (relative to the cost of conventional seed), farmers gained an average US $3.76 in extra income. In developing countries, the average return was $5.22 for each extra dollar invested in GM crop seed and in developed countries the average return was $3.00.

## Introduction

Crops containing genetically modified (GM) traits have been widely grown for 25 years and in 2020, the global area planted to crops was about 186 million hectares. The main crops using this technology are soybeans, maize, cotton, and canola, with GM traits present in just over 47% of the global area of these four crops in 2020.

Since the introduction of GM crop technology in the mid-1990s, there have been many analytical papers assessing the farm level economic and income impacts associated with the adoption of this technology. The author of this paper has undertaken some of these studies (eg, Brookes)^1^ and since 2005, has engaged in a regular (typically annual) exercise to identify, update, and aggregate the sum of these various studies, and where possible, to supplement them with new analysis. The aim of this has been to provide an up-to-date and as accurate as possible assessment of some of the key farm-level economic impacts associated with the global adoption of crops containing GM traits. It is also hoped the nalysis continues to contribute to understanding the impact of this technology and to facilitate more informed decision-making, especially in countries where crop biotechnology is currently not permitted.

This study updates the findings of earlier analysis into the global impact of GM crops since their commercial introduction in 1996 by extending analysis to include the years of 2019 and 2020. Previous analysis by the current author has been published in various journals, with the last analysis being Brookes and Barfoot 2020.^2^ The methodology and analytical procedures in this present discussion are unchanged so as to allow a direct comparison of the new with earlier data and analysis. Readers should note that some data presented in this paper are not directly comparable with data presented in previous analysis because the current paper also takes into account the availability of new data and analysis that may have not previously been available, including revisions to data for earlier years.

In order to save readers of this paper the chore of consulting the past papers for details of the methodology and arguments, these are included in full in this paper.

The analysis focuses on gross farm income effects because these are a primary driver of adoption amongst farmers (both large commercial and small-scale subsistence). It also quantifies the (net) production impact of the technology. The authors recognize that an economic assessment could examine a broader range of potential impacts (eg, on labor usage, household incomes, local communities, and economies). However, these are not included because undertaking such an exercise would add considerably to the length of the paper and an assessment of wider economic impacts would probably merit a separate assessment in its own right.

## Methodology

The report is based on detailed analysis of existing farm-level impact data for GM crops, much of which can be found in peer-reviewed literature. Most of this literature broadly refers to itself as “economic impact” literature and applies farm accounting or partial budget approaches to assess the impact of GM crop technology on revenue, the main variable costs of production (seed cost, crop protection, and weed control, use of labor and fuel/machinery) and gross farm income. Although primary data relating to impacts of commercial cultivation were not available for every crop, in every year and for each country, a substantial body of representative research and analysis is available and this has been used as the main basis for the analysis presented. The author has also undertaken his own analysis of the impact of some trait-crop combinations in some countries where the availability of published research is more limited (notably GM herbicide tolerant (HT) traits in North and South America). This analysis is mostly based on analysis of key input data, such as herbicide and insecticide usage/costs and seed variety use/costs.

The farm level economic impact of the technology varies widely, both between and within regions/countries. Therefore, the analysis is considered on a case by case basis, using average performance and impact recorded in different crop and trait combinations by the studies reviewed. Where more than one piece of relevant research (eg, on the impact of using a GM trait on the yield of a crop in one country in a particular year) has been identified, the findings used in this analysis reflect the authors assessment of which research is more likely to be reasonably representative of impact in the country as a whole and in a particular year. For example, there are many papers on the impact of GM insect resistant (IR) cotton in India in its early years of widespread usage. Few of these studies were reasonably representative of cotton growing across the country, with most based on small-scale, local, and therefore unrepresentative samples of cotton farmers. Only the reasonably representative research has been drawn on for use in this paper – readers should consult the references to this paper to identify the sources used.

This approach may still both, overstate, or understate, the impact of GM technology for some trait, crop and country combinations, especially in cases where the technology has provided yield enhancements. However, as impact data for every trait, crop, location, and year data is not available, the author has had to extrapolate available impact data from identified studies to years for which no data are available. In addition, if the only studies available took place several years ago, there is a risk that basing current assessments on such comparisons may not adequately reflect the nature of currently available alternative (non-GM seed or crop protection) technology. The author acknowledges that these factors represent potential methodological weaknesses. To reduce the possibilities of over/understating impact due to these factors, the analysis:

- Directly applies impacts identified from the literature to the years that have been studied. As a result, the impacts used vary in many cases according to the findings of literature covering different years. Examples where such data is available include the impact of GM insect resistant (IR) cotton: in India (see Bennett R et al. 2004,^3^ IMRB 2006^4^ and IMRB 2007,^5^) in Mexico (see Traxler et al. 2001^6^ and Monsanto/Bayer Mexico annual monitoring reports submitted to the Ministry of Agriculture in Mexico^7^) and in the USA (see Sankala & Blumenthal, (2003^8^ and 2006^9^) Mullins & Hudson 2004.^10^) Hence, the analysis takes into account variation in the impact of the technology on yield according to its effectiveness in dealing with (annual) fluctuations in pest and weed infestation levels;
- Uses current farm-level crop prices and bases any yield impacts on (adjusted – see below) current average yields. This introduces a degree of dynamic analysis that would, otherwise, be missing if constant prices and average yields identified in year-specific studies had been used;
- Includes changes and updates to the impact assumptions identified in the literature based on new papers, annual consultation with local sources (analysts, industry representatives, databases of crop protection usage and prices) and analysis of changes in crop protection product usage and prices and of seed varieties planted;
- Adjusts downwards the average base yield (in cases where GM technology has been identified as having delivered yield improvements) on which the yield enhancement has been applied. In this way, the impact on total production is not overstated.

Detailed examples of how the methodology has been applied to calculate the 2020 impacts are presented in Appendix A.

Other aspects of the methodology used to estimate the impact on direct farm income are, as follows:

- Where stacked traits have been used, the individual trait components were analyzed separately to ensure estimates of all traits were calculated. This is possible because the non-stacked seed has been (and in many cases continues to be) available and used by farmers and there are studies that have assessed trait-specific impacts;
- All values presented are nominal for the year shown and the base currency used is the US dollar. All financial impacts in other currencies have been converted to US dollars at prevailing annual average exchange rates for each year (source: United States Department of Agriculture Economics Research Service);
- The analysis focuses on changes in farm income in each year arising from impact of GM technology on yields, key costs of production (notably seed cost and crop protection expenditure) but also impact on costs, such as fuel and labor. Inclusion of these latter costs is more limited than the impacts on seed and crop protection costs because only a few of the papers reviewed have included consideration of such costs. In most cases, the analysis relates to impact of crop protection and seed cost only, crop quality (eg, improvements in quality arising from less pest damage or lower levels of weed impurities, which result in price premia being obtained from buyers) and the scope for facilitating the planting of a second crop in a season (eg, second crop soybeans in Argentina following wheat that would, in the absence of the GM HT seed, probably not have been planted). The farm income effect presented is, essentially, a gross margin impact (gross revenue minus variable costs of production) rather than a full net cost of production assessment. Through the inclusion of yield impacts and the application of actual (average) farm prices for each year, the analysis also indirectly takes into account the possible impact of GM crop adoption on global crop supply and world prices.

The paper also includes estimates of the production impacts of GM technology at the crop level. These have been aggregated to provide the reader with a global perspective of the broader production impact of the technology. These impacts derive from the yield impacts and the facilitation of additional soybean cropping within a season in South America. Details of how these values were calculated (for 2020) are shown in Appendix A.

## Results and Discussion

### Herbicide Tolerant (HT) Crops

GM HT crops were first grown widely in 1996 and in 2020 accounted for about 60% of the total GM crop plantings. The vast majority of these crops have been tolerant to the herbicide active ingredient glyphosate, although in the last few years the availability and use of crops tolerant to other herbicides has increased. The main impact of this technology has been to provide more cost-effective (less expensive) and easier weed control for farmers. Some users of this technology have also obtained higher yields from better weed control (relative to weed control obtained from conventional technology). The magnitude of these impacts varies by country and year, and the variation is due to several factors. These include the prevailing costs of different herbicides used in GM HT systems versus weed control practices in conventional (non-GM crops), which may include different/alternative herbicides to those used with GM HT crops and/or other forms of weed control (eg, hand or mechanical weeding), the mix and amounts of herbicides applied, the cost farmers pay for accessing the GM HT technology and the underlying levels of weed problems faced by farmers. Important factors affecting the level of cost savings achieved include:

- The mix and amounts of herbicides used on GM HT crops and conventional crops are affected by price and availability of herbicides. Herbicides used include both “older” products that are no longer protected by patents and newer “patent-protected” chemistry, with availability affected by commerical decisions of suppliers to market or withdraw products from markets and regulation (eg, changes to approval processes and the imposition of restrictions/bans). Prices also vary by year and country according to factors, such as exchange rates, costs of manufacture and distribution;
- The amount farmers pay for use of the technology varies by country and year. Pricing of technology (all forms of seed and crop protection technology, not just GM technology) varies according to the level of benefit that the technology providers perceive farmers are likely to derive from it. In addition, it is influenced by intellectual property rights (patent protection, plant breeders’ rights, and rules relating to use of farm-saved seed). In countries with weaker intellectual property rights, the cost of the technology tends to be lower than in countries where there are stronger rights. This issue is examined further below as it is a key factor determining take-up levels of the technology. Also, the HT technology available in 2020 is, in some countries, not the same as the technology available in the early years of adoption. As indicated above, in the first 15–20 years of widespread use of GM HT crop technology, crops tolerant to glyphosate dominated. In 2020, farmers, notably in North America now have the option of using seed tolerant to glyphosate plus other active ingredients like glufosinate, 2,4-D and dicamba. These forms of “stacked” herbicide tolerances are typically more expensive than the single herbicide tolerance traits of the early years of use;
- Where GM HT crops tolerant to glyphosate have been widely grown for a number of years, incidence of weed resistance to glyphosate have increased and become a major concern in many regions. This has been attributed to how glyphosate was used with GM HT crops in the early years of adoption. Due to its broad-spectrum, post-emergence activity and effectiveness in controlling weeds cheaply, it was often used as the sole method of weed control. This approach to weed control put tremendous selection pressure on weeds and contributed to the evolution of weed populations predominated by resistant individual weeds. It should, however, be noted that there are hundreds of resistant weed species confirmed in the International Survey of Herbicide Resistant Weeds (www.weedscience.com.^11^) Worldwide, there are 56 weed species that are currently resistant to glyphosate (accessed May 2022), compared to 169 weed species resistant to ALS herbicides (eg, chlorimuron ethyl commonly used in conventional soybean crops) and 87 weed species resistant to photosystem II inhibitor herbicides (eg, atrazine commonly used in maize production). It should also be noted that the problem of herbicide-resistant weeds has not been accelerated or exacerbated by the adoption of GM HT crops and the overall rate of newly confirmed herbicide-resistant weed species to all herbicide sites of action has slowed in the US since 2005 (Kniss, 2018.^12^) In addition, GM HT technology has played a major role in facilitating the adoption of no and reduced tillage production techniques in North and South America. This has also probably contributed to the emergence of weeds resistant to glyphosate and to weed shifts toward those weed species that are not well controlled by glyphosate. As a result, growers of GM HT crops have been, and continue to be advised to include other herbicides (with different and complementary modes of action) in combination with glyphosate in their weed management systems, even where instances of weed resistance to glyphosate may have not been found. In some cases, farmers may also be advised to revert to adopt cultural weed control practices such as plowing. This change in weed control practices also reflects the broader agenda of developing strategies across all forms of cropping systems to minimize and slow the potential for weeds developing resistance to existing weed control technology (eg, Norsworthy et al., 2012.^13^) In addition, in the last 5 years, the increasing array of new GM HT technology referred to above has offered farmers (notably in North America) crops that are tolerant to other herbicide active ingredients typically in combination with tolerance to glyphosate (and sometimes offering tolerance to three active ingredients). At the macro level, these changes have influenced the mix, total amount, cost, and overall profile of herbicides applied to GM HT crops. It has also resulted in the weed control costs associated with growing GM HT crops generally being higher in 2020 than in the early 2000s. However, as the analysis presented below shows, GM HT crops have continued to be popular with farmers as they offer important economic advantages for most users relative to the conventional (non-GM) alternative, either in the form of lower costs of production or higher yields (arising from better weed control). An important contributory factor to this (maintenance of cost saving advantage of GM HT systems versus conventional alternatives) is that many of the herbicides used in conventional production systems also face significant weed resistance issues themselves (in the mid 1990s this was one of the reasons why glyphosate tolerant soybeans were rapidly adopted, as glyphosate provided good control of these weeds). It is also important to note that if GM HT technology was no longer delivering net economic benefits, it is likely that farmers around the world would have significantly reduced their adoption of this technology in favor of conventional alternatives. The fact that GM HT global crop adoption levels have not fallen in recent years suggests that farmers must be continuing to derive important economic benefits from using the technology.

These points are further illustrated in the analysis below.

### GM HT Soybeans

The most common farm income gain arising from the use of this seed technology has derived from a reduction in the cost of production, mainly through lower expenditure on weed control (typically herbicides). These gains have averaged between $6/ha and $33.5 ha (Table 1).

**Table 1.** GM HT soybeans: summary of average gross farm-level income impacts 1996–2020 ($/hectare).

Country	Due to cost savings	Due to higher yields	Due to facilitation of second cropping
Romania	9	35.6	Not applicable
Argentina	22.6	Not applicable	294
Brazil	32.4	Not applicable	Not applicable
USA	33.5	80.8	Not applicable
Canada	20.6	81.5	Not applicable
Paraguay	16.6	Not applicable	311
Uruguay	22.5	Not applicable	Not applicable
South Africa	9.4	Not applicable	Not applicable
Mexico	12.2	27.8	Not applicable
Bolivia	6.0	61.2	Not applicable

Romania applies to 1999 to 2006 only

Higher yield impact for USA and Canada relates to higher yielding second generation GM HT soybeans from 2008

All values presented for cost savings are net after deduction of the cost of the technology. For further information, see appendix B

Where yield gains have occurred, from improvements in weed control, the average farm income gain has been higher, for example, in countries, such as Romania, Mexico, and Bolivia, where additional income gains of between $28/ha and $61/ha have been obtained. A second generation of GM HT soybeans also became available to commercial soybean growers in the US and Canada in 2009 which offered the same tolerance to glyphosate as the first generation of the GM HT seed (and the same cost saving) but with higher yielding potential. The realization of this potential is shown in the higher average gross farm income benefits (Table 1). GM HT soybeans have also facilitated the adoption of no and reduced tillage production systems in some countries, shortening the production cycle. This advantage has enabled many farmers in South America to plant a crop of soybeans immediately after a wheat crop in the same growing season. The second crop, additional to traditional “one crop” soybean production, has therefore added considerably to farm incomes and to the volumes of soybean production in countries, such as Argentina and Paraguay (Table 1).

In global terms, the farm-level impact of using GM HT technology in soybeans (excluding “Intacta soybeans” which have been grown widely in South America since 2013 and combine GM herbicide tolerance with GM insect resistance traits in soybeans: see below) was $4.12 billion in 2020. If the second crop benefits arising in Argentina and Paraguay are included the total is $5.64 billion. Cumulatively since 1996, the farm income benefit has been (in nominal terms) $57 billion ($74.65 billion if second crop gains in Argentina and Paraguay are included).

In terms of the total value of global soybean production in 2020, the additional farm income (inclusive of Argentine second crop gains) generated by the technology is equal to a value-added equivalent of 5.6%. These economic benefits should be placed within the context of a significant increase in the level of soybean production in the main GM adopting countries since 1996 (more than a doubling in the area planted in the leading soybean producing countries of the US, Brazil, and Argentina).

If it is assumed that all of the second crop soybean gains are effectively *de facto* “yield” gains, then of the total cumulative farm income gains from using GM HT soybeans, $43 billion (57%) is due to yield gains/second crop benefits and the balance, 43%, is due to cost savings. The important contribution of these second crop production gains (plus yield enhancements) to global supplies of soybeans is discussed further below (crop production impacts).

### GM HT and IR (Intacta) Soybeans

This combination of GM herbicide tolerance (to glyphosate) and insect resistance in soybeans was first grown commercially in 2013, in South America. Since then, the technology has been used on approximately 155.8 million hectares and contributed an additional $16 billion to gross farm income of soybean farmers in Argentina, Brazil, Paraguay, and Uruguay. Brazil accounted for 80% of the area planted to this seed and 82% of the total farm income gain.

**Figure 1.**
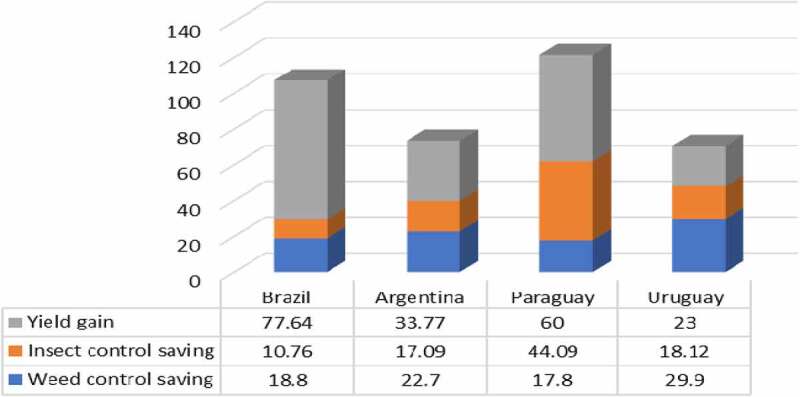
Breakdown of sources of income gain with intact soybeans by country 2013–2020 ($/ha). Additional details of the income gain components are available in Appendix B All values presented for cost savings are net after deduction of the cost of the technology

The average income gains over the eight years of adoption have been, respectively, $107.2/ha, $73.56/ha, $121.39/ha, and $70.68/ha in Brazil, Argentina, Paraguay, and Uruguay, through a combination of cost savings (decreased expenditure on herbicides and insecticides) and higher yields (see Fig. 1).

### GM HT Maize

**Figure 2.**
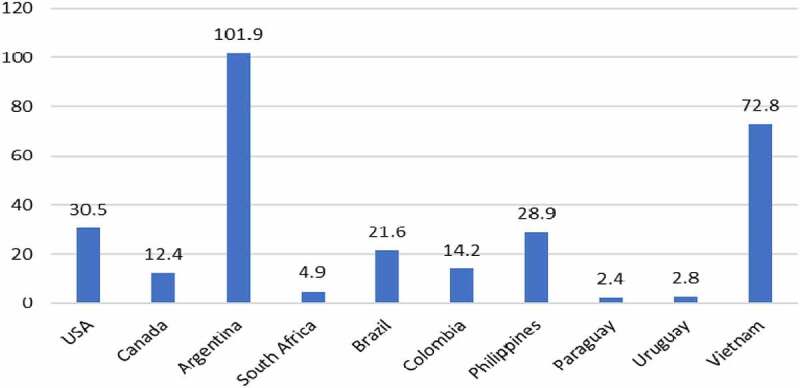
Average farm income gain from using GM HT maize by country: 1997–2020 ($/ha). Additional information relating these gains can be found in Appendices 1 and 2 All values presented are net after deduction of the cost of the technology

The adoption of GM HT maize has mainly resulted in lower costs of production, although yield gains from improved weed control have arisen in Argentina, Brazil, the Philippines, and Vietnam. As a result, the average level of income gain where cost savings have been the sole form of income gain has varied between $2.4/ha in Paraguay to 430.5/ha in the USA, rising up to over $100/ha (Argentina) when yield gains have been derived from improved weed control (Fig. 2).

In 2020, the total global farm income gain from using this technology was $1.55 billion with the cumulative gain over the period 1997–2020 being $20.2 billion. Within this, $7.8 billion (42%) was due to yield gains and the rest derived from lower costs of production.

### GM HT Cotton

A similar pattern of impact on farm income has occurred with the adoption of GM HT cotton since 1997. Most farmers have obtained cost savings, with some (in South America and Mexico) obtaining yield gains from improved levels of weed control. In 2020, the use of this technology delivered a gross farm income gain of about $134.8 million and in the 1997–2020 period, the total gross farm income benefit was $2.53 billion. Fifty eight percentage of these gains have been derived from cost savings, with the remaining 42% from yield gains.

Additional details relating to the nature of these income gain calculations in each adopting country are presented in Appendices 1 and 2.

### Other HT Crops

**Figure 3.**
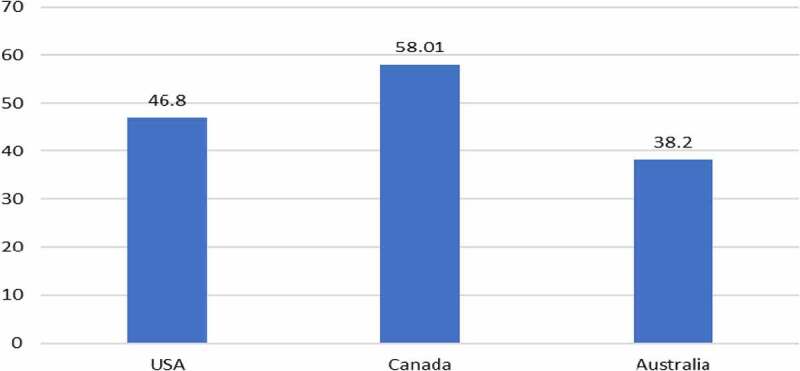
Average farm income gain from using GM HT canola by country: 1996–2020 ($/ha). Additional information relating these gains can be found in Appendices 1 and 2 All values presented are net after deduction of the cost of the technology

GM HT canola (tolerant to glyphosate or glufosinate) has been grown in Canada since 1996, in the USA since 1999 and in some states of Australia since 2008. The farm income impact in all three countries has been a combination of yield gains and some cost of production (weed control) savings, with the average income gain being within a range of $38/ha in Australia to $58/ha in Canada (Fig. 3). In 2020, the total global income gain from the adoption of GM HT technology in canola was $624 million and cumulatively since 1996, it has been $8.18 billion. Within this, 75% has been due to yield gains and the balance (23%) has been from cost savings. In terms of the total value of canola production in these three countries in 2020, the additional farm income generated by the technology is equal to a value-added equivalent of 5.9%.

GM HT (tolerant to glyphosate) sugar beet has been grown in the USA and Canada since 2008. The impact of using this technology has been to deliver a combination of yield gains and reductions in the cost of production (weed control). Since 2008, the average farm income gain has been about $130/ha, of which about $9/ha has derived from weed control cost savings and the balance of $121/ha has come from higher yields. In 2020, the total farm income benefit from using GM HT sugar beet in the USA and Canada was $77.3 million and since 2008, the cumulative income gain has been $755.3 million. Additional information relating to these farm income gains are presented in Appendices 1 and 2.

### Insect Resistant (GM IR) Crops

The main way in which these technologies have impacted on farm incomes has been through lowering the levels of pest damage and hence delivering higher yields. In addition, many farmers have made cost of production savings through less expenditure on insecticides and pest monitoring.

### GM IR Maize

**Figure 4.**
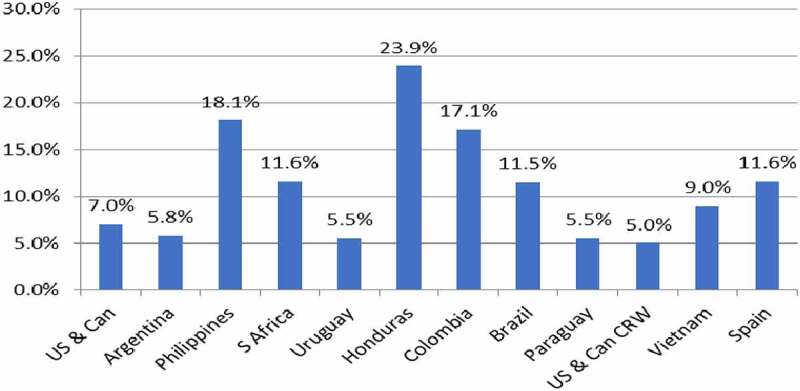
Average yield gains GM IR maize by country 1996–2020. Additional information relating these gains can be found in Appendices 1 and 2

This technology targets various stalk-boring pests that can cause significant yield losses to farmers around the world. In addition, in North America many GM varieties also contain traits that target the corn rootworm pest. The average yield gain performance of this technology since it was first used in 1996 is shown in (Fig. 4). Positive yield gains in the range of +5% to +23.9% have been recorded with an average yield gain across all user countries of +17.7%. The highest yield gains have occurred in developing countries, where conventional methods of pest control tend to be least effective (eg, reasons, such as poorly developed extension and advisory services, lack of access to finance to fund use of crop protection application equipment and products).

**Figure 5.**
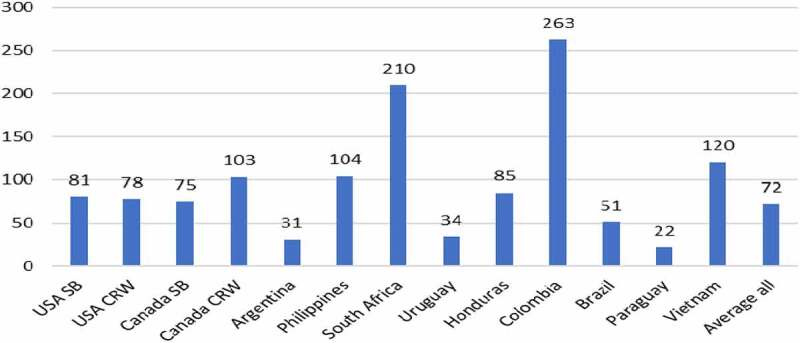
Average farm income gains GM IR maize by country 1996–2020. Additional information relating these gains can be found in Appendices 1 and 2 All values presented are net after deduction of the cost of the technology

The impact of these yield gains, coupled with some cost savings associated with less expenditure on insecticides and crop pest monitoring, on farm incomes is summarized in (Fig. 5). This shows that the average increase in farm income from using GM IR maize technology over the 1996–2020 period has been +$72/ha, within a range of +$22/ha and +$263/ha by user country. Aggregating these farm income gains, the total increase in farm income due to the use of GM IR maize between 1996 and 2020 has been $67.8 billion, with the increase in income in 2020 having been $3.7 billion.

### GM IR Cotton

**Figure 6.**
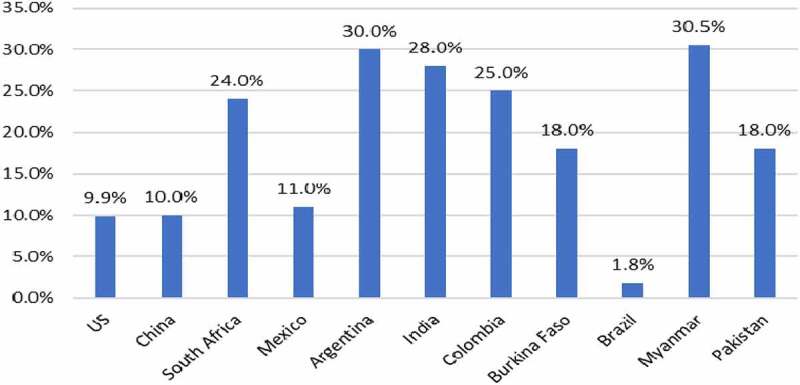
Average yield gains GM IR cotton by country 1996–2020. Additional information relating these gains can be found in Appendices 1 and 2

GM IR cotton seed technology helps farmers control the various bollworm/budworm pests that can are cause major problems for most cotton farmers. Before the availability of this technology, cotton crops in some countries (eg, China) were routinely sprayed 15–20 times per year in order to control these pests. With availability of GM IR seed technology the number and frequency of insecticide applications has fallen significantly to, typically, less than five, and focused on control of pests that the GM IR technology does not control (eg, sucking pests). The primary benefit of using this technology has been higher yields, with the average improvement in yield across all user countries between 1996 and 2020 having been +14.5%. Yield gains have been highest in developing countries (Fig. 6). In addition, most farmers have gained from reduced costs of production via notable reductions in the amount and frequency of insecticide applications. The only adopting country that has not experienced a yield improvement from using GM IR cotton technology has been Australia where the levels of boll and bud worm pests were relatively low before the first availability of GM IR technology because of effective use of intensive insecticide use programmes. The main benefit and reason for adoption of this technology in Australia has been the significant cost savings and the associated environmental gains from reduced insecticide use.

**Figure 7.**
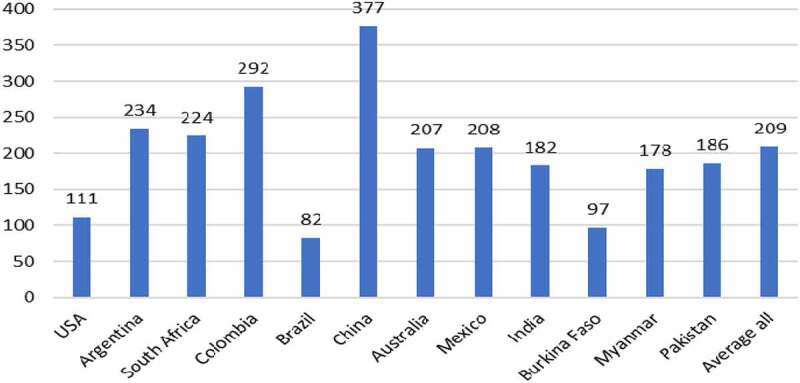
Average farm income gains GM IR cotton by country 1996–2020. Additional information relating these gains can be found in Appendices 1 and 2 All values presented are net after deduction of the cost of the technology

The average farm income gain from using GM IR cotton technology in the period 1996–2020 has been +$209/ha, with the highest levels of increase having been recorded in developing countries like China and Colombia (Fig. 7).

At the aggregate level, the global gross farm income gains from using GM IR cotton in 2020 was $3.8 billion and cumulatively since 1996, the gains have been $70.6 billion. Within this, 84% of the farm income gain has derived from yield gains (less pest damage) and the balance (16%) from reduced expenditure on crop protection (spraying of insecticides).

### GM Drought Tolerant Maize

Drought tolerant maize has been grown in parts of the US since 2014 and in 2020 was planted on 1.42 million hectares. Drawing on yield comparison data with other drought tolerant maize (varieties conveying drought tolerance that is not derived from GM technology) from field trials (source: Monsanto US Field Trials Network in the Western Great Plains,^14^) this suggests that the technology is providing users with a net yield gain of about 2.6% and a small cost saving in irrigation costs. After taking into consideration, the additional cost of the seed compared to non-GM drought tolerant maize, the average gross farm income gain (2014–2020) has been $17/ha. In 2020, this resulted to an aggregate farm income gain of $35.3 million and over the period 2014–2020, a total gain of $131.8 million.

### Aggregated (Global Level) Farm Income Impacts

GM crop technology has been used widely by many farmers for 25 years. It has helped farmers adapt their weed and pest control practices and enabled important improvements in yields to be realized. In turn, this has had a significant positive impact on global gross farm income. In 2020, this amounted to $18.8 billion, equivalent to having added 5.9% to the value of global production of the four main crops of soybeans, maize, canola, and cotton. Since 1996, gross farm incomes have increased by $261.3 billion.

At the country level, US farmers have been the largest beneficiaries of higher incomes, realizing $111 billion in extra income between 1996 and 2020. This is not surprising given that US farmers were first to make widespread use of GM crop technology and for many years the GM adoption levels in all four US crops have been in excess of 80%. Important farm income benefits ($71.6 billion) have occurred in South America (Argentina, Bolivia, Brazil, Colombia, Paraguay, and Uruguay), mostly from GM technology in soybeans and maize. GM IR cotton has also been responsible for an additional $53.6 billion additional income for cotton farmers in China and India.

In 2020, 55% of the farm income benefits were earned by farmers in developing countries. The vast majority of these gains have been from GM IR cotton and GM HT soybeans. Over the twenty-five years 1996–2020, the cumulative farm income gain derived by developing country farmers was $136.6 billion, equal to 52% of the total farm income during this period.

The average cost to farmers for accessing GM technology, across the four main crops, 1996–2020, was equal to 27% of the total value of technology gains. This is defined as the farm income gains referred to above plus the cost of the technology payable to the seed supply chain. Readers should note that the cost of the technology accrues to the seed supply chain, including sellers of seed to farmers, seed multipliers, plant breeders, distributors and the GM technology providers.

In developing countries, the total cost was equal to 19% of total technology gains compared with 33% in developed countries. Whilst circumstances vary between countries, the higher share of total technology gains accounted for by farm income in developing countries relative to developed countries reflects factors such as weaker provision and enforcement of intellectual property rights in developing countries and the higher average level of farm income gain per hectare derived by farmers in developing countries compared to those in developed countries.

In terms of investment, this means that for each extra dollar invested in GM crop seeds (relative to the cost of conventional seed), farmers gained an average US $3.76 in extra income. In developing countries, the average return was $5.22 for each extra dollar invested in GM crop seed and in developed countries the average return was $3.00.

Seventy-two percentage of the total income gain over the 25-year period derived from higher yields and second crop soybean gains with 28% from lower costs (mostly on insecticides and herbicides). In terms of the two main trait types, insect resistance and herbicide tolerance have accounted for 56% and 43.8%, respectively, of the total income gain (other traits of drought-resistant maize and virus-resistant papaya and squash accounted for the 0.2% balance). The balance of the income gain arising from yield/production gains relative to cost savings is changing as second-generation GM crops are increasingly adopted. In 2020, the split of total income gain was 91% from yield/production gains and 9% from cost savings.

### Crop Production Impacts

The positive yield impacts identified above plus the second soybean crop facilitation in South America have delivered important volumes to global production of maize, cotton, canola, and soybeans since 1996 (Table 2). The GM IR traits, used in maize and cotton, have accounted for 91.1% of the additional maize production and 98.2% of the additional cotton production. The small residual production gains have come from improvements in levels of weed control.

**Table 2.** Additional crop production arising from positive yield effects of GM crops (million tonnes).

	1996–2020	2020
Soybeans	330.35	33.48
Maize	594.58	47.9
Cotton	37.01	2.26
Canola	15.77	1.00
Sugar beet	1.87	0.15

Note: Sugar beet, US and Canada only (from 2008)

In soybeans, the second cropping in South America, enabled by increased use of NT/RT production systems, facilitated by GM HT technology has added 222.7 million tonnes to global soybean production, with Intacta soybeans added a further 44.4 million tonnes since 2013. The remaining additional GM-related soybean production has come from the second generation of GM HT soybeans grown in North American countries since 2008 and where the GM HT technology has enabled farmers to obtain higher yield via improved levels of weed control.

## Concluding Comments

Over the last 25 years, GM crop seed technology has helped many farmers to grow more food, feed, and fiber using fewer resources by reducing the damage caused by pests and better controlling weeds. The highest yield increases have occurred in developing countries and this has contributed to a more reliable and secure food supply base in these countries. In South America, HT technology has helped farmers adopt RT/NT production systems, shortening the time between planting and harvesting, allowing them the opportunity to grow an additional soybean crop after wheat in the same growing season.

With higher yields and less time and money spent managing pests and weeds, farmers have earned higher incomes. This has proven to be especially valuable for farmers in developing countries where, over the 25 year period 1996–2020, an average $5.22 was received for each extra dollar invested in biotech crop seeds.

The widespread use of GM crop technology has also contributed to changing agriculture’s land footprint by allowing farmers to grow more on existing land used for agricultural purposes, reducing the pressure to bring in new land into cultivation. For example, if world agriculture wanted to maintain global production of the four main crops in which GM seed technology has been widely used levels at 2020 levels, but without using the GM technology, this would require farmers to plant an additional 11.6 million ha of soybeans, 8.5 million ha of maize, 2.8 million ha of cotton, and 0.5 million ha of canola, an area (23.4 million ha in total) equivalent to the combined agricultural area of Philippines and Vietnam.

Nevertheless, in relation to the use of HT crops, over reliance on the use of glyphosate and the lack of crop and herbicide rotation by farmers, in some regions, has contributed to the development of weed resistance. In order to address this problem and maintain good levels of weed control, farmers have increasingly adopted more integrated weed management strategies incorporating a mix of herbicides, other HT crops and cultural weed control measures (in other words using other herbicides with glyphosate rather than solely relying on glyphosate, using HT crops, which are tolerant to other herbicides, such as dicamba, 2,4-D and glufosinate and using cultural practices such as mulching and reverting to plowing). This has added cost to the GM HT production systems relative to the costs of the early years of adoption over 20 years ago. Despite this, relative to the current conventional alternative, the GM HT technology continues to offer important economic benefits in 2020, as evidenced by the continued widespread use of this technology.

## Statistical Sources

AMIS Global/Kleffmann. Subscription-based data sources (derived from farmer surveys) on pesticide use. Kleffmann Gmbh, Ludinghausen, Germany. www.kleffmann.com

## Appendix A: Details Of Methodology As Applied To 2020 Farm Income CalculationsGM IR corn (targeting corn boring pests) 2020

**Table:**

Country	Area of trait (‘000 ha)	Yield assumption % change	Base yield (tonnes/ha)	Farm level price ($/tonne)	Cost of technology ($/ha)	Impact on costs, net of cost of technology ($/ha)	Change in farm income ($/ha)	Change in farm income at national level (‘000 $)	Production impact (‘000 tonnes)
US	27,367	+7	10.21	118	+24.82	+22.88	+61.53	+1,933,398	+21,669
Canada	1,135	+7	9.15	156	+25.1	+22.41	+77.20	+87,702	+727
Argentina	5,952	+5.5	7.07	139	+19.9	+19.9	+34.30	+204,159	+2,314
Philippines	683	+18	3.01	249	+40.16	+27.09	+107.93	+73,723	+370
South Africa	2,068	+10.6	5.42	179	+11.95	−1.2	+104.22	+215,496	+1,188
Spain	98	+12.6	11.40	211	+41.52	+34.33	+232.90	+22,860	+141
Uruguay	118	+5.5	5.74	176	+19.86	+19.86	+35.68	+4,199	+37
Honduras	32	+24	3.38	310	+100.0	+100.0	+151.46	+14,851	+26
Portugal	4	+12.5	7.85	228	+42.66	+44.66	+217.81	+918	+5
Brazil	18,045	+11.1	4.02	119	+57.18	+42.10	+11.14	+201,095	+8,053
Colombia	96	+16	5.37	220	+47.60	+5.80	+182.66	+17,581	+82
Paraguay	518	+5.5	5.34	139	+18.12	+16.12	+124.79	+12,853	+152
Vietnam	92	+10.2	4.78	235	+22.40	−15.28	+130.15	+11,974	+145

Impact on costs net of cost of technology = cost savings from reductions in pesticide costs, labor use, fuel use etc from which the additional cost (premium) of the technology has been deducted. For example (above) US cost savings from reduced expenditure on insecticides = -$15.88/ha, limited to an area equivalent to 10% of the total crop area (the area historically treated with insecticides for corn boring pests). This converted to an average insecticide cost saving equivalent per hectare of GM IR crop of -$1.94/ha. After deduction of the cost of technology (+$24.82/ha) is deducted to leave a net impact on costs of +$22.88 There are no Canadian-specific studies available, hence application of US study findings to the Canadian context (US being the nearest country for which relevant data is available)

**GM IR corn (targeting corn rootworm) 2020**

**Table:**

Country	Area of trait (‘000 ha)	Yield assumption % change	Base yield (tonnes/ha)	Farm level price ($/tonne)	Cost of technology ($/ha)	Impact on costs, net of cost of technology ($/ha)	Change in farm income ($/ha)	Change in farm income at national level (‘000 $)	Production impact (‘000 tonnes)
US	11,232	+5	10.21	118	+24.82	+14.47	+74.77	+839,793	+5,734
Canada	729	+5	9.15	156	+25.0	+8.85	+80.02	+58,338	+333

There are no Canadian-specific studies available, hence application of US study findings to the Canadian context (US being the nearest country for which relevant data is available)

**GM IR cotton 2020**

**Table:**

Country	Area of trait (‘000 ha)	Yield assumption % change	Base yield (tonnes/ha)	Farm level price ($/tonne)	Cost of technology ($/ha)	Impact on costs, net of cost of technology ($/ha)	Change in farm income ($/ha)	Change in farm income at national level (‘000 $)	Production impact (‘000 tonnes)
US	3,030	+10	0.824	1,343	+46.29	+14.48	+96.15	+291,323	+250
China	3,087	+10	1.805	2,696	+53.51	−25.11	+511.67	+1,519,789	+557
South Africa	15	+24	0.773	1,980	+20.62	−13.03	+354.21	+5,443	+3
Australia	267	Zero	2.22	1,969	+211.67	−165.69	+165.69	+44,259	Zero
Mexico	103	+10.3	1.431	1,399	+50.96	−35.83	+169.61	+17,524	+15
Argentina	441	+30	0.516	1,112	+21.25	−32.36	+206.03	+90,858	+68
India	12,220	+24	0.385	1,129	+10.83	+14.22	+124.26	+1,518,500	+1,129
Colombia	5	+20.7	0.80	1,392	+73.10	+13.17	+217.54	+1,004	+1
Brazil	1,169	+2.4	1.677	1,284	+25.24	−9.39	+60.54	+70,790	+47
Pakistan	2,090	+10	0.407	1,568	+9.07	−0.15	+63.94	+133,660	+85
Myanmar	214	+30	0.50	1,568	+20	+10.78	+224.83	+43,023	+32

Note Myanmar price based on Pakistan

**GM HT soybeans 2020 (excluding second crop soybeans – see separate table)**

**Table:**

Country	Area of trait (‘000 ha)	Yield assumption % change	Base yield (tonnes/ha)	Farm level price ($/tonne)	Cost of technology ($/ha)	Impact on costs, net of cost of technology ($/ha)	Change in farm income ($/ha)	Change in farm income at national level (‘000 $)	Production impact (‘000 tonnes)
US 1^st^ generation	3,758	Nil	3.38	348	+29.52	−15.66	+15.66	+58,839	Nil
US 2^nd^ generation	27,557	+8.9	3.143	348	+30.09	−15.09	+112.48	+3,099,585	+7,713
Canada 1^st^ generation	139	Nil	3.12	404	+47.22	−31.30	−31.30	+4,343	Nil
Canada 2^nd^ generation	1,616	+8.9	2.96	404	+52.53	−25.99	+132.27	+213,813	+426
Argentina	11,873	Nil	2.77	320	+2.5	−19.11	+19.11	+226,852	Nil
Brazil	12,986	Nil	3.55	299	+8.76	−32.68	+32.68	+424,357	Nil
Paraguay	1,787	Nil	2.88	278	+4.4	−15.10	+15.10	+26,987	Nil
South Africa	786	Nil	2.29	314	+1.13	−14.42	+14.42	+11,331	Nil
Uruguay	590	Nil	1.93	336	+2.5	−29.01	+29.01	+1,792	Nil
Bolivia	1,348	+15	2.17	88	+3.32	−5.96	+28.62	+38,586	+439

Price discount for GM soybeans relative to non GM soybeans in Bolivia of 2.7% – price for non GM soybeans was $91/tonne – price shown above is discounted

**GM IR/HT (Intacta) soybeans 2020**

**Table:**

Country	Area of trait (000’ ha)	Yield assumption % change	Base yield sucrose(tonnes/ha)	Farm level price: ($/tonne)	Cost of tech ($/ha)	Impact on costs, net of cost of tech ($/ha)	Change in farm income ($/ha)	Change in farm income at national level (‘000 $)	Production impact (‘000 tonnes)
Brazil	23,680	+9.4	3.36	299	+29.22	−20.35	+1114.74	+2,717,020	+7,467
Argentina	4,117	+7.1	2.76	340	+22.21	−14.00	+80.70	+332,210	+808
Paraguay	1,332	+11.5	2.99	278	+29.22	−31.70	+127.61	+169,931	+459
Uruguay	291	+7	1.89	336	+22.21	−24.62	+69.14	+20,096	+39

**GM HT corn 2020**

**Table:**

Country	Area of trait (‘000 ha)	Yield assumption % change	Base yield (tonnes/ha)	Farm level price ($/tonne)	Cost of technology ($/ha)	Impact on costs, net of cost of technology ($/ha)	Change in farm income ($/ha)	Change in farm income at national level (‘000 $)	Production impact (‘000 tonnes)
US	29,763	Nil	10.80	118	+24.82	−32.61	+32.61	+968,497	Nil
Canada	1,374	Nil	9.67	156	+31.61	−14.15	+14.15	+19,440	Nil
Argentina: as single trait	384	+3% con belt, +22% marginal areas	8.06 corn belt, 5.00 marginal areas	139	+19.86	−13.25	+33.68 corn belt, +153.23 marginal areas	+35,388	+291
Argentina: as stacked trait	5,888	+10.25	7.07	139	+19.90	−13.25	+87.69	+516,328	+4,267
South Africa	2.162	Nil	5.86	179	+10.46	−1.13	+1.13	+2,441	Nil
Philippines	680	+5	3.01	249	+40.16	+14.17	+23.31	+16,004	+103
Colombia	109	Zero	5.81	220	+23.16	−9.82	+9.82	+1,071	Nil
Brazil	16.459	+3	4.02	119	+28.16	+15.19	−0.73	−12,083	+1,993
Uruguay	129	Nil	6.00	176	+19.86	−13.25	+13.25	+1,706	Nil
Paraguay	472	Nil	5.56	139	+11.09	+0.36	+0.36	+170	Nil
Vietnam	92	+5	4.78	235	+11.03	+41.67	+97.87	+9,004	+22

Where no positive yield effect due to this technology is applied, the base yields shown are the indicative average yields for the crops and differ (are higher) than those used for the GM IR base yield analysis, which have been adjusted downwards to reflect the impact of the yield enhancing technology (see below)

Argentina: single trait. In the Corn Belt, it is assumed that 70% of trait plantings occur in this region and marginal regions account for the balance. In relation to stacked traits, the yield impact (+10.25%) is in addition to the yield 5.5% impact presented for the GM IR trait (above). In other words, the total estimated yield impact of stacked traits is +15.75%. The cost of the technology also relates specifically to the HT part of the technology (sold within the stack)

**GM HT cotton 2020**

**Table:**

Country	Area of trait (‘000 ha)	Yield assumption % change	Base yield (tonnes/ha)	Farm level price ($/tonne)	Cost of technology ($/ha)	Impact on costs, net of cost of technology ($/ha)	Change in farm income ($/ha)	Change in farm income at national level (‘000 $)	Production impact (‘000 tonnes)
US	3,153	Nil	0.897	1,343	+69.44	−6.05	+6.05	+18,961	Nil
S Africa	16	Nil	0.95	1,980	+11.7	−27.37	+27.37	+443	Nil
Australia	280	Nil	2.24	1,969	−54.5	−32.71	+32.71	+9,159	Nil
Argentina	450	Farm saved seed area nil Certified seed area +9.3%	0.668	1,22	+11.76 certified seed, nil farm saved seed	−5.84 certified seed, −17.6 farm saved seed	+ 84.98 certified seed, +17.6 farm saved seed	+15,742	+8
Mexico	146	+16	1.431	1,399	+37.8	−22.82	+296.46	+43,135	+33
Colombia	5	+4.0	0.88	1,392	+34.2	−29.47	+74.14	+358	+0.2
Brazil	1,226	+1.6	1.677	1,274	+25.96	−25.86	+38.32	+46,993	+33

Where no positive yield effect due to this technology is applied, the base yields shown are the indicative average yields for the crops and differ (are higher) than those used for the GM IR base yield analysis, which have been adjusted downwards to reflect the impact of the yield enhancing technology (see below)

Argentina: 30% of area assumed to use certified seed with 70% farm saved seed

**GM HT canola 2020**

**Table:**

Country	Area of trait (‘000 ha)	Yield assumption % change	Base yield (tonnes/ha)	Farm level price ($/tonne)	Cost of technology ($/ha)	Impact on costs, net of cost of technology ($/ha)	Change in farm income ($/ha)	Change in farm income at national level (‘000 $)	Production impact (‘000 tonnes)
US glyphosate tolerant	162	+2.0	1.99	379	+17.3	−6.75	+21.84	+3,527	+2
US glufosinate tolerant	526	+7.4	1.99	379	+17.3	+6.05	+50.55	+26,624	+23
Canada glyphosate tolerant	3,203	+2.0	2.14	470	+28.55	−30.41	+46.39	+148,588	+137
Canada glufosinate tolerant	4,784	+7.4	2.14	470	+4.17	−17.61	+87.85	+420,274	+758
Australia glyphosate tolerant	562	+8	1.84	408	+4.32	+0.89	+44.55	+25,062	+83

Baseline (conventional) comparison in Canada with herbicide tolerant (non GM) “Clearfield” varieties

**GM virus resistant crops 2020**

**Table:**

Country	Area of trait (ha)	Yield assumption % change	Base yield (tonnes/ha)	Farm level price ($/tonne)	Cost of technology ($/ha)	Impact on costs, net of cost of technology ($/ha)	Change in farm income ($/ha)	Change in farm income at national level (‘000 $)	Production impact (‘000 tonnes)
US Papaya	187	+17	12.21	968	+494	+494	+1,515	+283	+0.4
US squash	1,000	+100	19.6	575	+736	+736	+10,536	+10,536	+20

**GM herbicide tolerant sugar beet 2020**

**Table:**

Country	Area of trait (000’ ha)	Yield assumption % change	Base yield sucrose(tonnes/ha)	Farm level price equivalent (sucrose: $/tonne)	Cost of tech ($/ha)	Impact on costs, net of cost of tech ($/ha)	Change in farm income ($/ha)	Change in farm income at national level (‘000 $)	Production impact (‘000 tonnes)
US	462	+3.19	9.59	375	+148	−44.43	+159.20	+73,596	+141
Canada	17	+3.19	14.17	375	+148	−44.43	+214.01	+3,724	+3

**GM drought tolerant maize 2020**

**Table:**

Country	Area of trait (000’ ha)	Yield assumption % change	Base yield (tonnes/ha)	Farm level price: $/tonne	Cost of tech ($/ha)	Impact on costs, net of cost of tech ($/ha)	Change in farm income ($/ha)	Change in farm income at national level (‘000 $)	Production impact (‘000 tonnes)
US	1,421	+2.57	10.21	118	+6.19	+6.12	+24.87	+35,333	+299

**GM IR brinjal 2020**

**Table:**

Country	Area of trait (ha)	Yield assumption % change	Base yield (tonnes/ha)	Farm level price $/tonne	Cost of tech ($/ha)	Impact on costs, net of cost of tech ($/ha)	Change in farm income ($/ha)	Change in farm income at national level (‘000 $)	Production impact (‘000 tonnes)
Bangladesh	6,309	+19.6	9.76	1,913	Nil	−84.34	+692.79	+4,371	+12

**Second soybean crop benefits: Argentina**

An additional farm income benefit that many Argentine soybean growers have derived comes from the additional scope for second cropping of soybeans. This has arisen because of the simplicity, ease, and weed management flexibility provided by the (GM) technology which has been an important factor facilitating the use of no and reduced tillage production systems. In turn, the adoption of low/no tillage production systems has reduced the time required for harvesting and drilling subsequent crops and hence has enabled many Argentine farmers to cultivate two crops (wheat followed by soybeans) in one season. As such, the proportion of soybean production in Argentina using no or low tillage methods has increased from 34% in 1996 to 90% by 2005 and has remained at over 90% since then.

Farm-level income impact of using GM HT soybeans in Argentina 2020 (2): second crop soybeans

**Table:**

Year	Second crop area (million ha)	Average gross margin/ha for second crop soybeans ($/ha)	Increase in income linked to GM HT system (million $)
2020	5.3	269.80	1,422.5

Source & notes:

Crop area and gross margin data based on data supplied by Grupo CEO and the Argentine Ministry of Agriculture

The second cropping benefits are based on the gross margin derived from second crop soybeans multiplied by the total area of second crop soybeans

**Base yields used where GM technology delivers a positive yield gain**

In order to avoid over-stating the positive yield effect of GM technology (where studies have identified such an impact) when applied at a national level, average (national level) yields used have been adjusted downwards (see example below). Production levels based on these adjusted levels were then cross checked with total production values based on reported average yields across the total crop.

**Example: GM IR cotton (2020)**

**Table:**

Country	Average yield across all forms of production (t/ha)	Total cotton area (‘000 ha)	Total production (‘000 tonnes)	GM IR area (‘000 ha)	Conventional area (‘000 ha)	Assumed yield effect of GM IR technology	Adjusted base yield for conventional cotton (t/ha)	GM IR production (‘000 tonnes)	Conventional production (‘000 tonnes)
US	0.897	3,443	3,088	3,030	413	+10%	0.906	2,746	340
China	1.976	3,250	6,422	3,087	162	+10%	1.805	6,130	293

Note: Figures subject to rounding

## Appendix B: Trait Specific Summaries Of Impacts And SourcesGM HT Soybeans: Summary Of Average Gross Farm Level Income Impacts 1996-2020

**Table:**

Country	Cost of technology ($/ha)	Average gross farm income benefit (after deduction of cost of technology: $/ha)	Aggregate income benefit (million $)	Type of benefit	References
*1^st^ generation GM HT soybeans*					
Romania (to 2006 only)	50–60	104	44.6	Small cost savings of about $9/ha, balance due to yield gains of +13% to +31%	Brookes 2005^1^ Monsanto Romania^15^
Argentina	2–4	22.6 plus second crop benefits of 294	24,134.1	Cost savings plus second crop gains	Qaim and Traxler 2005^16^ Trigo and CAP 2006,^17^ Rodriguez et al 2021^18^ and updated from 2008 to reflect herbicide usage and price changes
Brazil	7–25	32.4	9,083.2	Cost savings	Parana Department of Agriculture 2004^19^ Galveo 2010^20–23^ and updated to reflect herbicide usage and price changes
US	15–57	33.5	14,064.7	Cost savings	Marra et al 2002^24^ Carpenter and Gianessi 2002^25^ Sankala and Blumenthal (2003^8^ and 2006^9^) Johnson and Strom 2008^26^ And updated to reflect herbicide price and common product usage
Canada	20–48	20.6	232.3	Cost savings	George Morris Center 2004^27^ and updated to reflect herbicide price and common product usage
Paraguay	4–10	16.6 plus second crop benefits of 311	1,522.6	Cost savings	Based on Argentina as no country-specific analysis identified. Impacts confirmed by industry sources and herbicide costs and usage updated 2009 onwards from herbicide survey data (AMIS Global/Kleffman/Kynetec)
Uruguay	2–4	22.5	271.8	Cost savings	Based on Argentina as no country-specific analysis identified. Impacts confirmed by industry sources and herbicide costs and usage updated 2009 onwards from herbicide survey data (AMIS Global/Kleffman/Kynetec)
South Africa	2–30	9.4	68.3	Cost savings	As there are no published studies available, based on data from industry sources and herbicide costs and usage updated 2009 onwards from herbicide survey data (AMIS Global/Kleffman/Kynetec)
Mexico	20–47	40	6.1	Cost savings plus yield impacts in range of −2% to +13%	Monsanto/Bayer annual monitoring reports submitted to Ministry of Agriculture and personal communications
Bolivia	3–4	67.2	957.1	Cost savings plus yield gain of +15%	Fernandez W et al 2009^28^
*2nd^t^ generation GM HT soybeans*					
US and Canada	30–67	114.3 (US) 102.1 (Can)	22,823.1 (US) 1,302.0 (Can)	Cost savings as first generation plus yield gains in range of +5% to +11%	As first-generation GM HT soybeans plus annual farm level survey data from Monsanto/Bayer USA
*Intacta soybeans*					
Brazil	29–53	107.2	13,200.7	Herbicide cost saving as 1^st^ generation plus insecticide saving $19/ha and yield gain +9% to +10%	Monsanto/Bayer Brazil pre commercial trials and post market (farm survey) monitoring, MB Agro 2013^29^
Argentina	19–53	73.6	1,535.0	Herbicide cost saving as 1^st^ generation plus insecticide saving $21/ha and yield gain +7% to +9%	Monsanto/Bayer Argentina pre commercial trials and post market monitoring surveys
Paraguay	29–53	121.4	1,147.6	Herbicide cost saving as 1^st^ generation plus insecticide saving $33/ha and yield gain +9% to +13%	Monsanto/Bayer Paraguay pre commercial trials and post market monitoring surveys
Uruguay	19–53	70.7	167.3	Herbicide cost saving as 1^st^ generation plus insecticide saving $19/ha and yield gain +7% to +9%	Monsanto/Bayer Uruguay pre commercial trials and post market monitoring surveys

Romania stopped growing GM HT soybeans in 2007 after joining the European Union, where the trait is not approved for planting. Mexico has not planted any GM HT soybeans since 2017 because of a government ban its cultivation

The range in values for cost of technology relates to annual changes in the average cost paid by farmers. It varies for reasons such as the price of the technology set by seed companies, exchange rates, average seed rates, and values identified in different studies

Intacta soybeans (HT and IR) first grown commercially in 2013

For additional details of how impacts have been estimated, see examples in Appendix A

AMIS Global/Kleffmann/Kynetec are subscription-based data sources (derived from farmer surveys) on pesticide use

References to Monsanto/Bayer Argentina, Brazil, Paraguay, and Uruguay as sources of data from pre-commericalisation trials and post market monitoring – this is unpublished data provided to the authors by these companies on a yearly basis covering seed premium, yield comparisons and cost of insecticide/number of insecticide treatment comparisons for Intacta crops versus conventional and GM HT (only) crops. The data derives from survey-based monitoring of sites growing each crop

GM HT maize: summary of average gross farm income impacts 1997–2020 (42,43)

**Table:**

Country	Cost of technology ($/ha)	Average gross farm income benefit (after deduction of cost of technology: $/ha)	Aggregate income benefit (million $)	Type of benefit	References
US	15–30	30.5	12,742.0	Cost savings	Carpenter and Gianessi 2002^25^ Sankala and Blumenthal (2003^8^ and 2006^9^) Johnson and Strom 2008^26^ Also updated annually to reflect herbicide price and common product usage
Canada	17–35	12.4	225.4	Cost savings	Monsanto/Bayer Canada (personal communications) and updated annually since 2008 to reflect changes in herbicide prices and usage
Argentina	13–33	101.9	4,562.3	Cost savings plus yield gains over 10% and higher in some regions	Personal communications from Monsanto/Bayer Argentina, Grupo CEO and updated since 2008 to reflect changes in herbicide prices and usage
South Africa	9–18	4.9	102.6	Cost savings	Personal communications from Monsanto/Bayer South Africa and updated since 2008 to reflect changes in herbicide prices and usage
Brazil	10–32	21.6	2,298.1	Cost savings plus yield gains of +1% to +7%	Galveo 2010^20–23^
Colombia	14–24	14.2	11.5	Cost savings	Mendez et al 2011,^30^ Brookes 2020^31^
Philippines	24–47	28.9	237.9	Cost savings plus yield gains of +5% to +15%	Gonsales 2009^32^ Monsanto/Bayer Philippines (personal communications) Updated since 2010 to reflect changes in herbicide prices and usage
Paraguay	11–17	2.4	7.4	Cost saving	Personal communications from Monsanto/Bayer Paraguay and AMIS Global/Kleffman/Kynetec – annually updated to reflect changes in herbicide prices and usage
Uruguay	6–17	2.8	2.5	Cost saving	Personal communication from Monsanto/Bayer Uruguay and AMIS Global/Kleffman/Kynetec – updated annually to reflect changes in herbicide prices and usage
Vietnam	11–28	72.8	23.0		Brookes 2017,^33^ Brookes and Dinh 2021^34^

The range in values for cost of technology relates to annual changes in the average cost paid by farmers. It varies for reasons such as the price of the technology set by seed companies, exchange rates, average seed rates, and values identified in different studies

For additional details of how impacts have been estimated, see examples in Appendix A

AMIS Global/Kleffmann/Kynetec are subscription-based data sources (derived from farmer surveys) on pesticide use

References to MonsantoBayer Argentina, Canada, South Africa, Philippines, Paraguay, and Uruguay as sources of data – this is unpublished data provided to the authors by these companies on a yearly basis covering seed premium and typical herbicide treatments used on GM HT and conventional crops

Reference to changes in herbicide prices and usage – author estimates drawing on AMIS Global/Kleffmann/Kynetec data and other similar database sources and extension services (eg, Ontario Ministry of Agriculture in Canada)

GM HT cotton summary of average gross farm income impacts 1997–2020

**Table:**

Country	Cost of technology ($/ha)	Average gross farm income benefit (after deduction of cost of technology: $/ha)	Aggregate income benefit (million $)	Type of benefit	References
US	13–82	16.2	1,172.4	Cost savings	Carpenter and Gianessi 2002^25^ Sankala and Blumenthal (2003^8^ and 2006^9^) Johnson and Strom 2008^26^ Also updated to reflect herbicide price and common product usage
South Africa	12–32	31.9	8.3	Cost savings	Personal communications from Monsanto/Bayer South Africa and updated since 2008 to reflect changes in herbicide prices and usage
Australia	32–82	28.2	145.4	Cost savings	Doyle et al 2003^35^ Monsanto/Bayer Australia (personal communications) and updated to reflect changes in herbicide usage and prices
Argentina	10–30	48.4	238.4	Cost savings and yield gain of +9%	Personal communications from Monsanto/Bayer Argentina, Grupo CEO and updated since 2008 to reflect changes in herbicide prices and usage
Brazil	26–54	53.1	397.3	Cost savings plus yield gains of +1.6% to +4%	Galveo 2010^20–23^
Mexico	29–79	297	546.7	Cost savings plus yield gains of +3% to +20%	Monsanto/Bayer Mexico annual monitoring reports submitted to the Ministry of Agriculture and personal communications
Colombia	34–96	63.7	18.9	Cost savings plus yield gains of +4% (note −5% in first year of adoption – 2008/09)	Monsanto/Bayer Colombia annual personal communications, Brookes 2020^31^

The range in values for cost of technology relates to annual changes in the average cost paid by farmers. It varies for reasons such as the price of the technology set by seed companies, exchange rates, average seed rates, the nature, and effectiveness of the technology (eg, second generation “Flex” cotton offered more flexible and cost-effective weed control than the earlier first generation of HT technology) and values identified in different studies

For additional details of how impacts have been estimated, see examples in Appendix A

Note negative yield impact of yield in first year of adoption mainly due to technology not being available in leading and locally adapted varieties

References to Monsanto/Bayer Argentina, Australia, South Africa, and Colombia as sources of data – this is unpublished data provided to the authors by these companies on a yearly basis covering seed premium and typical herbicide treatments used on GM HT and conventional crops

Reference to Monsanto/Bayer Mexico annual monitoring reports. These are unpublished, annual monitoring of crop reports that the company is required to submit to the Mexican Ministry of Agriculture, as part of post market monitoring requirements. This provides data on seed premia, cost of weed control and production and yields for GM HT cotton versus conventional to a regional level

Reference to changes in herbicide prices and usage – author estimates drawing on AMIS Global/Kleffmann/Kynetec data and other similar database sources and extension services (eg, New South Wales Department of Agriculture in Australia)

Other GM HT crops summary of average gross farm income impacts 1996–2020

**Table:**

Country	Cost of technology ($/ha)	Average farm income benefit (after deduction of cost of technology: $/ha)	Aggregate income benefit (million $)	Type of benefit	References
*GM HT canola*					
US	12–33	47	419.8	Mostly yield gains of +1% to +12% (especially Invigor canola)	Sankala and Blumenthal (2003^8^ and 2006^9^) Johnson and Strom 2008^26^ And updated to reflect herbicide price and common product usage
Canada	2–32	58	7,566.2	Mostly yield gains of +3% to +12% (especially Invigor canola)	Canola Council 2001^36^ Gusta et al 2011^37^ and updated to reflect herbicide price changes and seed variety trial data (on yields)
Australia	9–41	38.2	156.8	Mostly yield gains of +12% to +22% (where replacing triazine tolerant canola) but no yield gain relative to other non GM (herbicide tolerant canola)	Monsanto Australia 2009,^38^ Fischler and Tozer 2009^39^ and Hudson and Richards 2014^40^
*GM HT sugar beet*					
US and Canada	130–151	130	755.3	Mostly yield gains of +3% to +13%	Kniss 2008^41^ Khan 2008^42^ Jon-Joseph et al 2010^43^ Annual updates of herbicide price and usage data

In Australia, one of the most popular type of production has been canola tolerant to the triazine group of herbicides (tolerance derived from non GM techniques). It is relative to this form of canola that the main farm income benefits of GM HT (to glyphosate) canola has occurred

InVigor’ hybrid vigor canola (tolerant to the herbicide glufosinate) is higher yielding than conventional or other GM HT canola and derives this additional vigor from GM techniques

The range in values for cost of technology relates to annual changes in the average cost paid by farmers. It varies for reasons such as the price of the technology set by seed companies, exchange rates, average seed rates, and values identified in different studies

For additional details of how impacts have been estimated, see examples in Appendix A

References to Monsanto Australia as a source of data – this is unpublished data provided to the authors by this company on a yearly basis covering seed premium and typical herbicide treatments used on GM HT and conventional crops

Reference to changes in herbicide prices and usage – author estimates drawing on AMIS Global/Kleffmann/Kynetec data

Average (%) yield gains GM IR cotton and maize 1996–2020

**Table:**

	Maize insect resistance to corn boring pests	Maize insect resistance to rootworm pests	Cotton insect resistance	References
US	7.0	5.0	9.9	Carpenter and Gianessi 2002^25^ Marra et al 2002^24^ Sankala and Blumenthal (2003^8^ and 2006^9^) Hutchison et al 2010^44^ Rice 2004^45^ Mullins and Hudson 2004^10^
China	N/a	N/a	10.0	Pray et al 2002^46^
South Africa	11.0	N/a	24.0	Gouse et al (2005^47^, 2006a^48^ and 2006b^49^) Van der Wald 2010^50^ Ismael et al 2002^51^ Kirsten et al 2002^52^ James 2003^53^
Honduras	23.9	N/a	N/a	Falk Zepeda et al (2009^54^ and 2012^55^)
Mexico	N/a	N/a	11.0	Traxler and Godoy-Avila S 2001^6^ Monsanto Mexico annual cotton monitoring reports^7^
Argentina	5.8	N/a	30.0	Trigo 2002^56^ Trigo and Cap 2006^17^ Rodriguez et al 2021^18^ Qaim and De Janvry (2002^57^ and 2005^58^) Elena 2001^59^
Philippines	18.1	N/a	N/a	Gonsales 2005^60^ Gonsales et al 2009^32^ Yorobe 2004^61^ Ramon 2005^62^
Spain	11.6	N/a	N/a	Brookes (2003^63^ and 2008^64^) Gomez-Barbero, Barbel M A and Rodriguez-Corejo 2008^65^ Riesgo et al 2012^66^
Uruguay	5.6	N/a	N/a	As Argentina (no country-specific studies available and industry sources estimate similar impacts as in Argentina)
India	N/a	N/a	28.0	Bennett et al 2004^3^ IMRB (2006^4^ and 2007^5^) Herring and Rao 2012^67^
Colombia	17.1	N/a	25.0	Mendez et al 2011^30^ Zambrano et al 2009^68^ Brookes 2020^31^
Canada	7.0	5.0	N/a	As US (no country-specific studies available and industry sources estimate similar impacts as in the US)
Burkina Faso	N/a	N/a	18.0	Vitale J et al 2008,^69^ Vitale J 2010^70^
Brazil	11.5	N/a	1.8	Galveo 2009^20–23^,2015^71^ Monsanto Brazil 2008^72^
Pakistan	N/a	N/a	18.0	Nazli et al 2010,^73^ Kouser and Qaim 2013^74^,2014^75^
Myanmar	N/a	N/a	30.5	USDA 2011^76^
Australia	N/a	N/a	Nil	Doyle 2005^77^ James 2002^78^ CSIRO 2005^79^ Fitt 2001^80^
Paraguay	5.5	N/a	Not available	As Argentina (no country-specific studies available and industry sources estimate similar impacts as in Argentina)
Vietnam	9.0	N/a	N/a	Brookes 2017,^33^ Brookes and Dinh 2020^34^

N/a = not applicable

Not included in table – also IR brinjal grown in Bangladesh an average yield gain 2013/14 to 2018/19 of +17.3%

7% yield gain in the US associated with performance of GM IR technology to suppress corn boring pests includes benefits to non GM maize growers via areawide suppression of pests, estimated by Hutchison et al (2020^44^) to account for 60% of the gross gains in the US between 1996 and 2009

Reference to Monsanto/Bayer Mexico annual monitoring reports. These are unpublished, annual monitoring of crop reports that the company is required to submit to the Mexican Ministry of Agriculture, as part of post market monitoring requirements. This provides data on seed premia, cost of pest control and production and yields for GM IR cotton versus conventional to a regional level

GM IR maize performance in Uruguay and Paraguay. Industry sources consulted for using Argentina impact data as a suitable proxy for impact in these countries include Monsanto/Bayer Argentina, Uruguay and Paraguay, Argenbio (Argentine Biotechnology Association) and Trigo E (Grupo CEO)

GM IR crops: average gross farm income benefit 1996–2020

**Table:**

Country	GM IR maize: cost of technology: $/ha	GM IR maize (income benefit after deduction of cost of technology: $/ha)	Aggregate income benefit GM IR maize (million $)	GM IR cotton: cost of technology: $/ha	GM IR cotton (income benefit after deduction of cost of technology: $/ha)	Aggregate income benefit GM IR cotton (million $)
US	17–32 IRCB, 22–42 IR CRW	81 IRCB, 78 IR CRW	51,762.3	26–58	111	7,069.1
Canada	17–26 IRCB, 22–42 IR CRW	75 IRCB 103 IR CRW	2,042.1	N/a	N/a	N/a
Argentina	10–33	31	1,901.4	21–86	234	1,244.6
Philippines	30–47	104	851.0	N/a	N/a	N/a
South Africa	9–17	94	2,568.7	14–50	224	74.9
Spain	17–51	210	371.9	N/a	N/a	N/a
Uruguay	11–33	34	46.8	N/a	N/a	N/a
Honduras	100	85	52.4	N/a	N/a	N/a
Colombia	30–49	263	214.8	73–112	292	100.0
Brazil	44–69	51	7,856.7	25–52	82	435.1
China	N/a	N/a	N/a	38–60	377	26,268.6
Australia	N/a	N/a	N/a	85–299	207	1,135.6
Mexico	N/a	N/a	N/a	48–75	208	407.4
India	N/a	N/a	N/a	11–54	182	27,370.4
Burkina Faso	N/a	N/a	N/a	51–54	97	204.6
Myanmar	N/a	N/a	N/a	17–20	178	552.8
Pakistan	N/a	N/a	N/a	9–15	186	5,531.3
Paraguay	16–20	22	71.0	N/a	N/a	N/a
Vietnam	22–42	120	37.9		N/a	
**Average across all user countries**		**72**			**209**	

GM IR maize all are IRCB unless stated (IRCB = insect resistance to corn boring pests), IRCRW = insect resistance to corn rootworm

The range in values for cost of technology relates to annual changes in the average cost paid by farmers. It varies for reasons such as the price of the technology set by seed companies, the nature, and effectiveness of the technology (eg, second generation “Bollgard” cotton offered protection against a wider range of pests than the earlier first generation of “Bollgard” technology), exchange rates, average seed rates, and values identified in different studies.

Average across all countries is a weighted average based on areas planted in each user country

n/a = not applicable

Sources – as above yields table

## References

[1] Brookes G. The farm level impact of using roundup ready soybeans in Romania. Agbioforum. 2005; 8(4): 235–41. www.agbioforum.org/the-farm-level-impacts-of-herbicide=tolerant-soybeans-in-Romania/

[2] Brookes G, Barfoot P. (2020) GM crop technology use 1996-2018: farm income and production impacts. GM Crops & Food. 2020;11(4):242–61. doi:10.1080/21645698.2020.1779574. 32706314 PMC7518751

[3] Bennett R, Ismael Y, Kambhampati U, Morse S. Economic impacts of GM cotton in India. AgBioforum. 2004;7:96–100.

[4] IMRB. Socio-economic benefits of bollgard and product satisfaction (in India). Mumbai (India): IMRB International; 2006.

[5] IMRB. Socio-economic benefits of bollgard and product satisfaction (in India). Mumbai (India): IMRB International; 2007.

[6] Traxler G, Godoy-Avila S. Transgenic cotton in Mexico. Agbioforum. 2004;7:57–62.

[7] Monsanto Comercial Mexico. Official reports to Mexican ministry of agriculture of the (year) cotton crop, unpublished papers for 2008, 2009, 2010, 2011, 2012, 2013, 2014, 2015, 2016

[8] Sankala S, and Blumenthal E. Impacts on US agriculture of biotechnology-derived crops planted in 2003- an update of eleven case studies. Washington, DC: NCFAP; 2003. www.ncfap.org.

[9] Sankala S, and Blumenthal E. Impacts on US agriculture of biotechnology-derived crops planted in 2005- an update of eleven case studies. Washington, DC: NCFAP; 2005. www.ncfap.org.

[10] Mullins W, and Hudson J. Bollgard II versus bollgard sister line economic comparisons, 2004 beltwide cotton conferences. USA: San Antonio; 2004 Jan.

[11] Weedscience.org. Website of international survey of herbicide resistant weeds. www.weedscience.org

[12] Kniss AR. Genetically engineered herbicide-resistant crops and herbicide-resistant weed evolution in the United States. Weed Science. 2018;66(2):260–73. doi:10.1017/wsc.2017.70.

[13] Norsworthy JK, Ward SM, Shaw DR, Llewellyn RS, Nichols RL, Webster TM, Bradley KW, Frisvold G, Powles SB, Burgos NR, Witt WW, and Barrett M. et al. Reducing the risk of herbicide resistance: best management practices and recommendations. Weed Science. 2012;31–62. Herbicide Resistant Weeds Special.

[14] Monsanto US field trials network in the western great plains. https://monsanto.com/products/learning-centers/gothenburg-learning-center/

[15] Romania M. Unpublished results of farmer survey amongst soybean growers in 2006 –. published. 2007.

[16] Qaim M, Traxler G. Roundup ready soybeans in Argentina: farm level & aggregate welfare effects. Agricultural Economics. 2005;32(1):73–86. doi:10.1111/j.0169-5150.2005.00006.x.

[17] Trigo E, Cap E. Ten years of GM crops in Argentina agriculture. ArgenBio. 2006. http://argenbio.org/biblioteca/Ten_Years_of_GM_Crops_in_Argentine_Agriculture_02_01_07.pdf.

[18] Rodriguez A, Rossi S, George N, Trigo E. 25 years of genetically modified crops in argentine agriculture. Argenbio. 2021. www.argenbio.org/transgenicos25.

[19] Parana Department of Agriculture. Cost of production comparison: biotech and conventional soybeans, in USDA GAIN report, 2004, BR4629 of 11 November 2004. www.fas.usad.gov/gainfiles/200411/146118108.pdf

[20] Galveo A. Farm survey findings of impact of insect resistant corn and herbicide tolerant soybeans in Brazil. Brazil: Celeres; 2010. www.celeres.co.br.

[21] Galveo A. Farm survey findings of impact of GM crops in Brazil. Brazil: Celeres; 2011. www.celeres.co.br.

[22] Galveo A. Farm survey findings of impact of GM crops in Brazil 2012. Celeres: Brazil. www.celeres.co.br

[23] Galveo A. Farm survey findings of impact of GM crops in Brazil 2015. Celeres: Brazil. www.celeres.co.br

[24] Marra M, Pardey P, Alston J. The pay-offs of agricultural biotechnology: an assessment of the evidence. Washington (USA): International Food Policy Research Institute; 2002.

[25] Carpenter J, Gianessi L. Agricultural biotechnology: updated benefit estimates. Washington (USA): National Centre for Food and Agricultural Policy (NCFAP; 2002.

[26] Johnson S, and Strom S. Quantification of the impacts on US agriculture of biotechnology-derived crops planted in 2006. Washington, DC: NCFAP; 2008. www.ncfap.org.

[27] George Morris Centre. Economic & environmental impacts of the commercial cultivation of glyphosate tolerant soybeans in Ontario, 2004, unpublished report for Monsanto Canada

[28] Fernandez W, Paz R, Zambrano P, Zepeda JF. GM soybeans in Bolivia, 2009, paper presented to the 13th ICABR conference, Ravello, Italy, June 2009

[29] MB Agro. Intacta soybeans: an economic view of the benefits of adopting the new technology, 2014 report commissioned by Monsanto Brazil

[30] Mendez K, Chaparro Giraldo A, Reyes Moreno G, Silva Castro C. Production cost analysis and use of pesticides in the transgenic and conventional maize crop in the valley of San Juan (Colombia). GM Crops. 2011 June-Dec;2(3):163–68. 2011. 22008311 10.4161/gmcr.2.3.17591

[31] Brookes G. Genetically modified (GM) crop use in Colombia: farm level economic and environmental contributions. GM Crops & Food. 2020;11(3):140–53. doi:10.1080/21645698.2020.1715156. 32008444 PMC7518743

[32] Gonsales L. Modern biotechnology and Agriculture: a history of the commercialisation of biotechnology maize in the Philippines. Los Banos (Philippines): Strive Foundation; 2009. 978-971-91904-8-6.

[33] Brookes G. The potential socio-economic and environmental impacts from adoption of corn hybrids with biotech trait/technologies in Vietnam. UK: PG Economics; 2017.

[34] Brookes G, Dinh DX. The impact of using genetically modified (GM) corn/maize in Vietnam: results of the first farm-level survey. GM Crops & Food. 2021;12(1):71–83. doi:10.1080/21645698.2020.1816800. 32997586 PMC7657581

[35] Doyle B. The performance of roundup ready cotton 2001-2002 in the Australian cotton sector. Armidale (Australia): University of New England; 2003.

[36] Canola Council of Canada. An agronomic & economic assessment of transgenic canola. Canola Council (Canada); 2001. www.canola-council.org.

[37] Gusta MS, Belcher S, K PP, Castle D. Economic benefits of GM HT canola for producers. AgBioForum. 2011;14: 1:1–12.

[38] Monsanto Australia. Survey of herbicide tolerant canola licence holders 2008

[39] Fischer J, Tozer P. Evaluation of the environmental and economic impact of roundup ready canola in the Western Australian crop production system, 2009, Curtin University of Technology Technical Report 11/2009

[40] Hudson D, Richards R. Evaluation of agronomic, environmental, economic and co-existence impacts following the introduction of GM canola in Australia 2010-2012. Agbioforum. 2014;17:1–12.

[41] Kniss A. Comparison of conventional and glyphosate resistant sugarbeet the year of commercial introduction in Wyoming. Journal of Sugar Beet Research. 2010;47(3):127–34. doi:10.5274/jsbr.47.3.127.

[42] Khan M. Roundup ready sugar beet in America. British Sugar Beet Review Winter. 2008;76:16–19.

[43] Jon-Joseph AQ, Sprague CL. Weed management in wide-and narrow-row glyphosate resistant sugar beet. Weed Technology. 2010;24(4):523–28. doi:10.1614/WT-D-10-00033.1.

[44] Hutchison W, Burkness EC, Mitchel PD, Moon RD, Leslie TW, Fleicher SJ, Abrahamson M, Hamilton KL, Steffey KL, Gray ME, et al. Area-wide suppression of European corn borer with Bt maize reaps savings to non-bt maize growers. Science. 2010;330(6001):222–25.www.sciencemag.org. 20929774 10.1126/science.1190242

[45] Rice M. Transgenic rootworm corn: assessing potential agronomic, economic and environmental benefits. Plant Health Progress. 2004;10:094/php-2001-0301-01-RV.

[46] Pray C, Hunag J, Hu R, Roselle S. Five years of Bt cotton in China – the benefits continue. The Plant Journal. 2002;31(4):423–30. doi:10.1046/j.1365-313X.2002.01401.x. 12182701

[47] Gouse M, Pray C, Kirsten J, Schimmelpfennig D. A GM subsistence crop in Africa: the case of Bt white maize in S Africa. Int Journal Biotechnology. 2005;7(1/2/3):84–94. doi:10.1504/IJBT.2005.006447.

[48] Gouse M, Piesse J, Thirtle C. Output & labour effect of GM maize and minimum tillage in a communal area of Kwazulu-Natal. Journal of Development Perspectives. 2006;2:192–207.

[49] Gouse M, Pray C, Kirsten J, Schimmelpfennig D. Three seasons of insect resistant maize in South Africa: have small farmers benefited. AgBioforum. 2006;9:15–22.

[50] Van der Weld W. Final report on the adoption of GM maize in South Africa for the 2008/09 season, South African Maize Trust, 2009

[51] Ismael Y, Bennet R, Morse S. Benefits of bt cotton use by smallholder farmers in South Africa. Agbioforum. 2002;5:1–5.

[52] Kirsten J, Gouse M. Bt cotton in South Africa: adoption and the impact on farm incomes amongst small-scale and large-scale farmers, ICABR conference, Ravello, Italy 2002

[53] James C. Global review of commercialized transgenic crops 2002: feature Bt maize. ISAAA;29:2003.

[54] Falck Zepeda J, Sanders A, Trabanino R, Medina O, Batallas-Huacon R. Small ‘resource poor’ countries taking advantage of the new bio-economy and innovation: the case of insect protected and herbicide tolerant corn in Honduras, 2009, paper presented to the 13th ICABR conference, Ravello, Italy, June 2009

[55] Falck Zepeda J, Sanders A, Trabanino R, Medina O, Batallas-Huacon R. Caught between Scylla and Charybdis: impact estimation issues from the early adoption of GM maize in Honduras. Agbioforum. 2012;15:138–51.

[56] Trigo E. Genetically modified crops in Argentina agriculture: an opened story. Buenos Aires (Argentina): Libros del Zorzal; 2002.

[57] Qaim M, De Janvry A. Bt cotton in Argentina: analysing adoption and farmers’ willingness to pay, 2002, American Agricultural Economics Association Annual Meeting, California,

[58] Qaim M, De Janvry A. Bt cotton and pesticide use in Argentina: economic and environmental effects. Environment and Development Economics. 2005;10(2):179–200. doi:10.1017/S1355770X04001883.

[59] Elena M. Economic advantages of transgenic cotton in Argentina, INTA, 2006, cited in Trigo and CAP 2006,

[60] Gonsales L. Harnessing the benefits of biotechnology: the case of Bt corn in the Philippines. Laguna (Philippines): Strive Foundation; 2005. 971-91904-6-9.

[61] Yorobe J. Economics impact of Bt corn in the Philippines, 2004, Paper presented to the 45th PAEDA Convention Querzon City

[62] Ramon G. Acceptability survey on the 80-20 bag in a bag insect resistance management strategy for Bt corn. Biotechnology Coalition of the Philippines (BCP). 2005.

[63] Brookes G. The farm level impact of using Bt maize in Spain, ICABR conference paper 2003, Ravello, Italy. Also on www.pgeconomics.co.uk

[64] Brookes G. The benefits of adopting GM insect resistant (Bt) maize in the EU: first results from 1998-2006. International Journal of Biotechnology. 2008;10(2/3):148–66. doi:10.1504/IJBT.2008.018351.

[65] Gomez-Barbero M, Barbel J, Rodriguez-Cerezo E. Adoption and performance of the first GM crop in EU agriculture: Bt maize in Spain. 2008. JRC, EU Commission. Eur 22778. http://www.jrc.ec.europa.eu 10.1038/nbt0408-384 18392015

[66] Riesgo L, Areal F, Rodriguez-Cerezo E. How can specific market demand for non-GM maize affect the profitability of Bt and conventional maize? A case study for the middle Ebro Valley, Spain. Spanish Journal of Agricultural Research. 2012;10(4):867–76. doi:10.5424/sjar/2012104-448-11.

[67] Herring R, Rao C. On the ‘failure of Bt cotton’: analysing a decade of experience. Economic and Political Weekly. 2012;47:5/5/2012.

[68] Zambrano P. Insect resistant cotton in Colombia: impact on farmers, paper presented to the 13th ICABR conference, 2009, Ravello, Italy

[69] Vitale J, Glick H, Greenplate J, Traore O. The economic impact of 2nd generation Bt cotton in West Africa: empirical evidence from Burkina Faso. International Journal of Biotechnology. 2008;10(2/3):167–83. doi:10.1504/IJBT.2008.018352.

[70] Vitale J. Impact of bollgard II on the socio economic and health welfare of smallholder cotton farmers in Burkina Faso: results of the 2009 field survey14^th^ ICABR conference, Ravello, Italy, June 2010

[71] Galveo A. Unpublished data on first survey findings of impact of insect resistant corn (first crop) in Brazil. Brazil: Celeres; 2009. www.celeres.co.br.

[72] Brazil M. Farm survey of conventional and Bt cotton growers in Brazil. unpublished. 2007.

[73] Nazli H, Sarker R, Meilke K, Orden D. Economic performance of Bt cotton varieties in Pakistan. Conference paper at the Agricultural and Applied Economics Association 2010 AAEA, CAES and WACA Joint Annual Meeting, Denver, USA

[74] Kouser S, Qaim M. Bt cotton, damage control and optimal levels of pesticide use in Pakistan. Environment and Development Economics. 2014;19(6):704–23. doi:10.1017/S1355770X1300051X.

[75] Kouser S, Qaim M. Valuing financial, health and environmental benefits of Bt cotton in Pakistan. Agricultural Economics. 2013;44(3):323–35. doi:10.1111/agec.12014.

[76] USDA. New technologies aiding Burmese cotton farmers, GAIN report BM 0025 of 14th January 2011

[77] Doyle B. The performance of ingard and bollgard ii cotton in Australia during the 2002/2003 and 2003/2004 seasons. Armidale (Australia): University of New England; 2005.

[78] James C. 2002. Global review of commercialized transgenic crops 2001: feature Bt cotton. ISAAA;(26).

[79] CSIRO. The cotton consultants Australia 2005 bollgard II comparison report, CSIRO, Australia

[80] Fitt G. Deployment and impact of transgenic Bt cotton in Australia, reported in James C (2001). Global Review of Commercialised Transgenic Crops: 2001 Feature: Bt Cotton, ISAAA.

